# The background assimilation effect: Facial emotional perception is affected by surrounding stimuli

**DOI:** 10.1177/20416695231190254

**Published:** 2023-08-29

**Authors:** Yujie Wu, Haojiang Ying

**Affiliations:** Department of Psychology, 12582 Soochow University, Suzhou, China

**Keywords:** emotion valence, face perception, assimilation effect, summary statistics

## Abstract

The perception of facial emotion is not only determined by the physical features of the face itself but also be influenced by the emotional information of the background or surrounding information. However, the details of such effect are not fully understood. Here, the authors tested the perceived emotion of a target face surrounded by stimuli with different levels of emotional valence. In Experiment 1, four types of objects were divided into three groups (negative, unpleasant flowers and unpleasant animals; mildly negative (neutral), houses; positive, pleasant flowers). In Experiment 2, three groups of surrounding faces with different social–emotional valence (negative, neutral, and positive) were formed with the memory of affective personal knowledge. The data from two experiments showed that the perception of facial emotion can be influenced and modulated by the emotional valence of the surrounding stimuli, which can be explained by assimilation: the positive stimuli increased the valence of a target face, while the negative stimuli comparatively decreased it. Furthermore, the neutral stimuli also increased the valence of the target, which could be explained by the social positive effect. Therefore, the process of assimilation is likely to be a high-level emotional cognition rather than a low-level visual perception. The results of this study may help us better understand face perception in realistic scenarios.

The ability to recognize facial emotion correctly and effortlessly is an essential aspect of interpersonal communication. It has been repeatedly suggested that the perception of facial emotion is not only determined by the physical feature of a face (e.g., Calder & Young, 2005; Ekman, 1992) but also by many other external factors: vocal expressions (e.g., Cowen et al., 2019), body postures (e.g., de Gelder et al., 2015), cultural background (e.g., Masuda et al., 2008), etc. Therefore, emotional perception is a complex process, whose foundation depends on our ability to derive and integrate information from a variety of cues across different modalities.

In many social scenarios, faces hardly appear in isolation. They appear with other objects and faces in particular environments that provide a rich source of contextual information (Oliva & Torralba, 2007) in many cases. In recent years, more and more studies begin to investigate the contributions of visual context to facial emotional perception. For example, Chen and Whitney (2021) found that visual context could induce emotional perception as effective as face and body. By using a set of highly variable natural scenes, Righart and de Gelder (2008a, 2008b) found that the emotional information of background scenes could affect the perceived emotion of a face. Aviezer et al. (2012) also found that static images of ambient facial expressions (in high arousal states) cannot be recognized in terms of emotional valence until more context is given to participants, and similar results were also found by Van den Stock et al. (2007). Thus, the emotional information from the context is an essential and indispensable element of emotional recognition of a face.

These findings provoke an interesting question: how could the contextual stimuli affect the perceived emotional information of the target? Existing studies investigating the perception of facial attractiveness in group context may help us answer this question. It has been repeatedly found that the surrounding faces would affect the attractiveness of a target face: a face would be more attractive to look at with surrounding faces than being alone (Burns et al., 2021; Walker & Vul, 2014). Ying et al. (2019) suggested that this effect (at least) comprises a “social positive effect” and a “contrastive process.” The “social positive effect” means that being viewed within a group per se would increase the attractiveness of the target face. Interestingly, this effect is also present when the surrounding stimuli are not human faces. For instance, Carragher et al. (2019) found that when a target face was surrounded by houses instead of faces, the attractiveness of the target face can be improved significantly from when it was alone. Therefore, mere presence of surrounding stimuli, whatever they are faces or objects, could make the target face look more attractive to look at. Considering there is a strong connection between the emotion and attractiveness (Elias et al., 2017; Oosterhof & Todorov, 2008), one may wonder whether it is possible for facial emotion perception to be affected by the mere presence of surrounding stimuli in the background (regardless of their emotional information).

Note that, although some facial expressions are commonly linked with a certain valence, but it is not the case at all conditions. For instance, the facial expression of smile may not equal to the emotion of happiness at all social conditions (e.g., Ambadar et al., 2009; Shore & Heerey, 2011). Thus, the same expression may be perceived as with emotional valence of either positive (POS), neutral (NEU), or negative (NEG) at different conditions. Therefore, in this study, we focus on the perceived emotional valence of a face rather than the facial expression (which is more likely to be subjective to different interpretations).

Many researchers agreed that the facial emotion would be recognized faster and comparatively more accurately if the context is emotionally congruent with the facial emotion (Aviezer et al., 2012; Righart & de Gelder, 2006). But the direction of this context effect on emotion is still in debate. Whether the effect of these contextual emotional stimuli on the perceived emotion of the target face is an “assimilation effect” or a “contrast effect” shall be further clarified to better understand the context effect. To explore this contextual effect on face emotional perception, in Experiment 1, we chose four kinds of objects with different emotional valences (negative, neutral, positive) as flankers. If contextual stimuli with positive valence would boost the emotion of the target, while negative flankers would inhibit the perceived facial emotion, then this would be consistent with our “assimilation effect” hypothesis. If the results are reversed, this would be consistent with the “contrast effect” hypothesis.

What's more, to further explore the mechanism behind context effect on emotion, we conducted the Experiment 2 with group faces. Previously, many researchers have found facial expressions can convey information regarding individuals’ emotions and social intentions (Todorov et al., 2009), which is of great importance to making inferences about the social characteristics of a face (e.g., Engell et al., 2010). However, there is little research focusing on the effect of social information (e.g., personal affective knowledge) on facial emotion perception, especially in the context. This is understandable because the time required for an individual to read a social behavior statement far exceeds the time required for the target face to be presented. Also, Hsieh et al. (2021) found that even when surrounding stimuli were removed from the testing screen, the attractiveness of the target face and body could be affected in the “memory” condition. Therefore, through the memory task, we expect that faces that were not originally emotional will acquire the social–emotional valence of the corresponding behavior. We try to investigate whether surrounding faces with social–emotional valence formed from affective personal knowledge on memories could affect the emotional perception of the target face. If so, the process of assimilation between the emotional valence of target face and surrounding stimuli is likely to be a high-level emotional cognition rather than a low-level visual perception.

## Experiment 1: Surrounding Non-face Stimuli Affect the Perceived Emotional Valence of the Target Face

Many studies found that the emotional information of the background would affect the perceived emotion of a face (e.g., by Righart & de Gelder, 2008a, 2008b). Here, we conducted the first experiment online to validate this effect with a modified experimental design to conceptually replicate the findings and probe the mechanism behind it.

### Methods

#### Participants

Fifty subjects (37 females and 13 males; mean age 21.2 years) from Soochow University, with normal or corrected-to-normal vision, participated in this experiment. Apart from one of the authors (Y. W.), the other 49 subjects were naïve to the purpose of the experiment. From the post-hoc power analysis (with α-value of 0.05, *η*^2^_p_ = 0.174, G*Power 3.1.9.7), we found this sample size yielded a high power 1−*β* ≈ 1. All participants provided informed consent, with ethics approved by the Ethics Committee at Soochow University, China.

#### Stimuli

##### Surrounding Images

**Figure 1.**
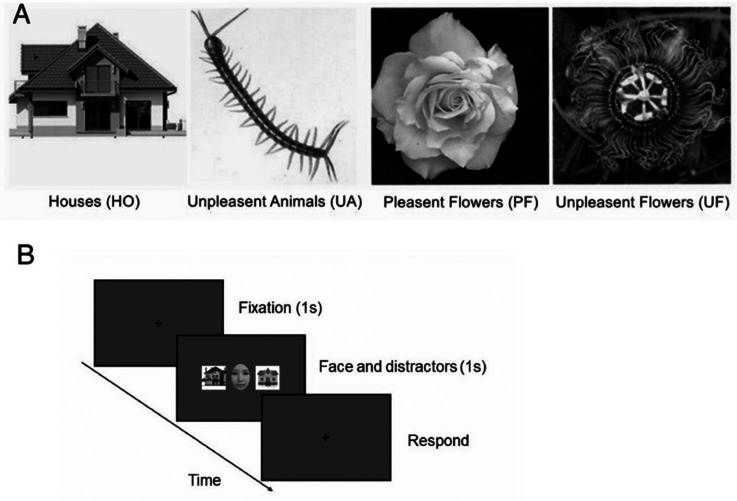
(A) The examples of four categories of surrounding images. (B) The sequence of an HO trial, where the target face is surrounded by two houses.

We selected 40 images from four categories for this experiment (Figure 1A): 10 images of houses (HO), 10 images of unpleasant animals (UA), 10 images of pleasant flowers (PF), and 10 images of unpleasant flowers (UF). The images of house were selected from the Microsoft Bing image. The rest were chosen from the computational vision at Caltech-101 database (Li et al., 2022). All these images (including the stimuli and the background) were turned into grayscaled, and luminance was equalized by the Picture Factory (Picosmos Inc.). All images were the size of 255 × 255 pixels. As illustrated in Figure 1, some stimuli with light colors (e.g., flowers) are presented with dark backgrounds, while some stimuli with dark colors (e.g., the house) are presented with light backgrounds. To better present the stimuli (e.g., avoiding its contour to be confused with background color), we chose to compromise the consistency of the background luminance. However, by doing so, we could preserve the integrity of the stimuli presentation. Therefore, we turned each image (as a whole) into grayscaled versions with equalized luminance.

To better select the stimuli, we asked another group of 30 volunteers (25 females and 5 males) to rate the emotional valence of these objects (paradigm adapted from Rhodes & Jeffery, 2006; Ying et al., 2020). During this test, 40 images of four categories were presented individually in randomized sequences. Volunteers were asked to rate these images for the valence on an 8-point scale (1 for most negative, 4.5 for neutral, and 8 for most positive). Results showed that HO (*M* = 4.73, *SD* = 1.559), PF (*M* = 5.63, *SD* = 1.721), UF (*M* = 2.29, *SD* = 1.297), and UA (*M* = 1.75, *SD* = 1.137). However, to simplify the current online experiment and shorten the possible duration, we randomly selected four images for each category. The mean emotions of the stimuli were HO (*M* = 4.07, *SD* = 1.494), PF (*M* = 5.36, *SD* = 1.544), UF (*M* = 2.13, *SD* = 1.107), and UA (*M* = 1.842, *SD* = 1.045).

We chose four kinds of common objects. House is one kind of common stimuli widely used by many researchers. For instance, Carragher et al. (2019) found that being surrounded by houses would affect the face perception just as that of surrounding faces. Since the animals (“bugs” in layman's term) are usually with severely negative emotional valence (NEG) (e.g., the centipede and spider), we select them as negative stimuli. While the house and centipede are indeed different in their shapes and meanings, they might be processed by different mechanisms, and we further selected different kinds of flowers as experimental material: pleasant flowers [e.g., the *Paeonia suffruticosa Andr* (Peony), as Righart and de Gelder (2008a, 2008b) did] and unpleasant flowers (e.g., the *Hydnora africana*).

Note that, unlike the pleasant and unpleasant flowers, we did not select the “positive” version of the unpleasant animals. We did so for two reasons. Firstly, the “animals” here, in layman's term, are “bugs”; however, centipede and spider are not insects (the scientific term for “bug”). Therefore, we called the stimuli animals, but the readers shall be aware that they are not typical animals like cat (*Felis catus*). Secondly, such kind of animals is typically unpleasant, and it is almost impossible to find positive versions of them with a similar shape (unlike the pleasant and unpleasant flowers that are all round-shaped): we can’t find a lovely and pleasant version of spider that satisfies every participant.

##### Target Faces

All the female face stimuli were chosen from the Taiwanese Facial Expression Image Database (TFEID; Chen & Yen, 2007). We first generated 21 faces with different levels of emotional expression from the same averaged identity (of all female faces from the database), morphed from sad (coded as 0% of happiness) to neutral (coded as 50% of happiness) to happy (coded as 100% of happiness) in 5% steps (via WebMorph; DeBruine, 2018). To fit psychometric functions, we selected seven target faces for this experiment, with 0%, 20%, 30%, 50%, 70%, 80%, and 100% of happiness [modified form the research conducted by Ying and Xu (2017)].

#### Apparatus

This experiment was conducted online at http://www.faceresearch.cn/. Participants were asked to complete it by using the computer alone.

#### Procedure

There were five experimental blocks in this experiment: (1) the baseline condition with only the target face, (2) the HO condition where the target face was surrounded by two houses, (3) the PF condition where the target face was surrounded by two pleasant flowers, (4) the UF condition where the target face was surrounded by two unpleasant flowers, and (5) the UA condition where the target face was surrounded by two unpleasant animals. The order of trials was randomized for each participant.

During the experiment, each trial presented one of the seven emotional faces and two of the four objects in each category randomly. However, to minimize any possible fatigue effect of the participants, we tried to limit the number of test trials. There were 42 trials (we did not swap the locations of the two surrounding stimuli) for each of the four experimental and one baseline conditions, for a total of 210 trials. The completion time was approximately 10 min. Thus, each emotional face was repeated six times, and each surrounding image was repeated twice within each group. Since the task consisted of categorizing facial emotions, participants were required to focus centrally on the face–object stimulus. The target face was presented in the center of the screen, and two flankers were presented on the left and right sides of the target face. Each trial commenced with a 1-s interval (Figure 1B). Then, the target face appeared with (or without) the two flankers for 1 s. After they disappeared, the participants were asked to report whether the target face is positive or negative [pressing “1” (on the keyboard) for positive and pressing “2” (on the keyboard) for negative]. This variation of two-AFC (two-alterative forced choice) has been wildly used to study the perception of faces.

### Results

**Figure 2.**
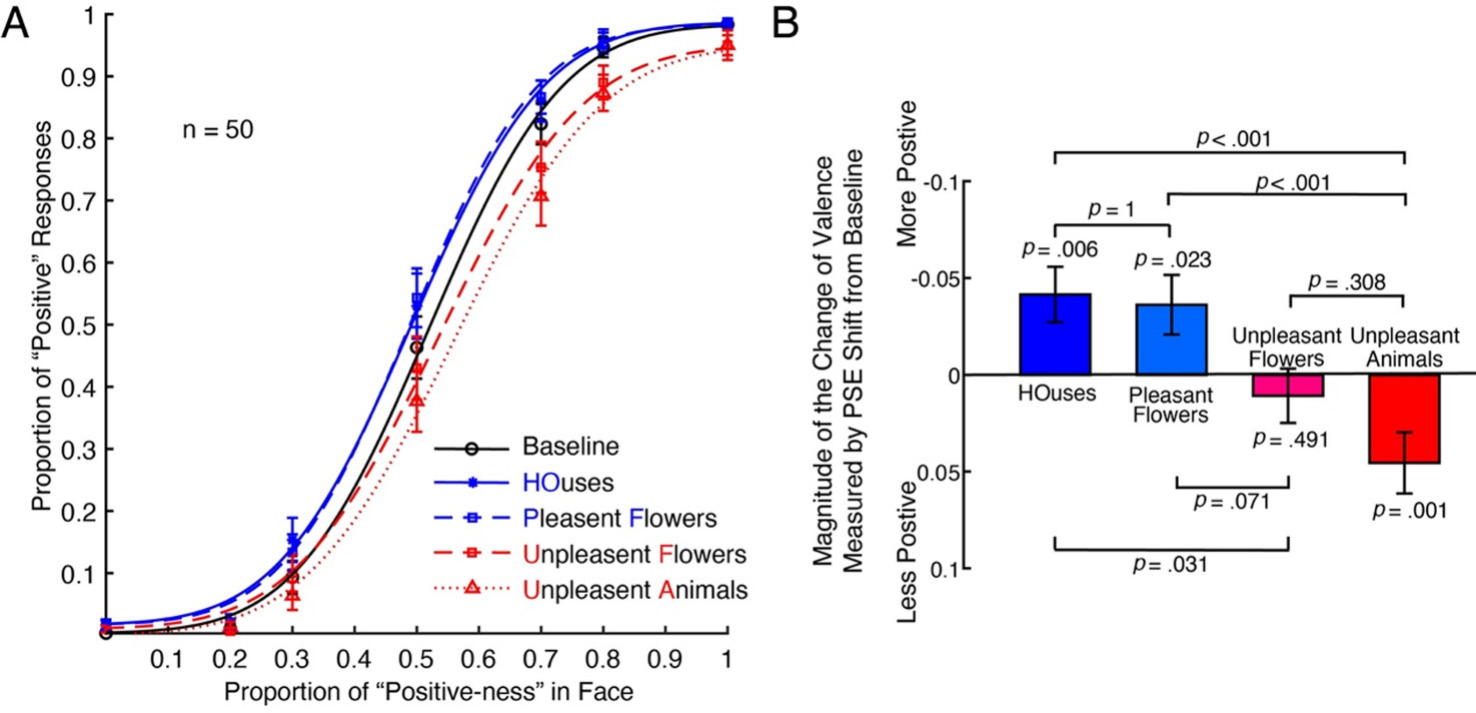
The effect of emotional surrounding stimuli on the target face (Experiment 1). (A) Psychometric functions from 50 subjects under the following conditions: Baseline, no surrounding stimuli (black solid line); houses, the face surrounded by two houses (blue solid line) looks more positive and the effect is significant; pleasant flowers (PF), the face surrounded by two pleasant flowers (blue-dashed line) looks more positive and the effect is significant; unpleasant flowers (UF), the face surrounded by two unpleasant flowers (red-dashed line) looks more negative and the effect is close to significant; unpleasant animals (UA), the face surrounded by two unpleasant animals (red solid line) looks more negative and the effect is dramatically significant. (B) The summary of all participant's data. For both HO and PF conditions, the perceived emotional valence of the target face has been significantly increased, while for UA conditions, the perceived emotional valence of the target face has been significantly decreased. For the UF condition, the perceived emotional valence of the target face has been decreased, but it's not significant. In this case, based on the calculation formula of point of subjective equality (PSE) shift, the PSE shift values shall be negative when the target face is more positive and vice versa. To make the figure easier to interpret and compare, we flip the y-axis in the same way as Burns et al. (2021). The pairwise comparisons here were with Bonferroni corrections.

At first, we plotted the mean psychometric functions of all subjects in Figure 2A. The black psychometric curve was the baseline condition without surrounding stimuli. The solid blue lines represented houses (HO), and the dashed blue lines represent pleasant flowers (PF). The dashed red lines represented unpleasant flowers (UF), and the dotted red lines represented unpleasant animals (UA). As indicated in the leftward shift of the psychometric function relative to the baseline condition, for both HO and PF conditions, the target face was biased to be positive, while for the UF and UA conditions, the target face was biased to be negative. Thus, the perceived emotional valence of the target face changed in line with the mean emotional valance of surrounding stimuli.

To test whether different emotional valences of the surrounding items would affect the perceived emotion of the target face, we conducted one sample *t*-test on the change of the proportion of positiveness (compared to baseline) of each category of surrounding images (Figure 2B). A positive value means a rightward shift of the psychometric curve (fewer positive responses) relative to the baseline. A negative value means a leftward shift of the psychometric curve (more positive responses) relative to the baseline. The results suggested that at the HO [*M*_HO_ = −0.042, *SEM* = 0.014, *t*(49) = −2.898, *p* = .006, Cohen's *d* = −0.410] and the PF [*M*_PF_ = −0.036, *SEM* = 0.015, *t*(49) = −2.345, *p* = .023, Cohen's *d* = −0.332] conditions, the perceived emotional valence of the target faces was biased to be more positive. At the UF [*M*_UF_ = 0.011, SEM = 0.016, *t*(49) = −0.694, *p* = .491, Cohen's *d* = 0.098], the emotion of the target face is not significantly affected, but at the UA [*M*_UA_ = 0.047, SEM = 0.014, *t*(49) = −3.379, *p* = .001, Cohen's *d* = 0.478] condition, the perceived emotional valence of the target faces was biased to be more negative.

To qualify the differences among the four categories of images, we conducted a repeated-measures ANOVA. The results suggested that the four categories were significantly different [Greenhouse–Geisser correction *F*(2.31,10.32) = 10.322, *p* < .001, *η*^2^_p_ = 0.174]. To further compare the differences among the four groups, we conducted pairwise comparisons (with Bonferroni corrections). The results suggested that differences between HO and PF conditions were not significant (*p* = 1), differences between HO and UF conditions were significant (*p *= .031), differences between HO and UA conditions were dramatically significant (*p *< .001), differences between PF and UF conditions were close but not significant (*p *= .071), and differences between PF and UA conditions were also significant (*p *< .001). However, differences between UA and UF conditions were not significant (*p *= .308).

At last, we conducted a Bayesian factor analysis to further investigate the “difference” between the HO and PF conditions: the results suggested that the data favors the notion that there is good evidence the two conditions were indeed close to each other (*BF*_01_ = 6.057 > 3), which means the effects of houses and pleasant flowers on target face are similar. However, the same analysis indicated that the “difference” between the PF and UF conditions was not strong enough, which seems to be not perfect, requiring further investigation (*BF*_01_ = 0.644 < 3. Also, the “difference” between the UF and UA conditions was not strong enough (*BF*_01_ = 0.638 < 3), requiring further validation in future research.

### Discussion

The results of the first experiment suggested that the perceived emotional valence of a face can be influenced by the emotional information of surrounding non-face objects. Moreover, the magnitudes of the emotion change were modulated by the emotional valence of the surrounding stimuli. When the surrounding stimuli were of negative emotional valence (unpleasant flowers and unpleasant animals), one would perceive the face as more negative than baseline, where the directions of change are consistent with their levels of emotional valence. When the stimuli were of positive emotional valence (POS) (pleasant flowers), it is more likely for one to perceive the target face as positive. Moreover, when the stimuli were of mildly negative emotional valence (houses), the target face was also perceived as more positive than baseline. This could be explained by a variation of the social positive effect: simply being in a group makes a target face more positive. What's more, the differences between PF and UA conditions were significant, which is consistent with our assimilation hypothesis. Moreover, the differences between different kinds of flowers (UF, PF) were close to significant, which further suggested that it is the valence of the flower, but not the flower shape per se (low-level visual feature of flowers in general) alters the perception of the target face. However, the differences between HO and PF conditions were not significant, and one of the possible reasons is that the emotional valences of house and pleasant flowers are not strong enough, which needs further research. Therefore, the impact of surrounding stimuli on the target face is consistent with the existence of both a social positive effect and a process of assimilation between the emotional valences of the surrounding objects and the target face.

## Experiment 2: The Emotional Valence of Surrounding Faces Affects the Perceived Emotional Valence of the Target Face

To further validate the findings from Experiment 1, we tried to conceptually replicate the experiment using a group of neutral expression faces with “attached artificial” emotional information. Using faces as stimuli would help us better control the low-level visual features of the stimuli, which is a potential limitation of the Experiment 1. Previously, Todorov et al. (2007) used behavioral and neural measurements and found that viewing a face and behavior descriptive sentence pair would induce consistent and long-lasting emotional memory of that face. Here in Experiment 2, we extend this method to conceptually validate our findings from Experiment 1: testing the perception of emotional valence of a face with neutral emotion surrounded by faces which had been associated with a category of behavior descriptive sentence with either positive, neutral, or negative emotional valence.

### Methods

#### Participants

Forty subjects (33 females and 7 males; mean age 21.23 years) from Soochow University, with normal or corrected-to-normal vision, participated in this experiment. All subjects were naïve to the purpose of the experiment. We selected this sample size because it is comparable to previous research examining ensemble coding via face adaptation aftereffects (we doubled the sample size from Ying et al., 2019). From the post-hoc power analysis (with α-value of 0.05, *η*^2^_p_ = .286, G*Power 3.1.9.7), we found this sample size yield a high power 1−*β* = 0.96. All participants provided informed consent, with ethics approved by the Ethics Committee at Soochow University, China.

#### Stimuli

##### Behavior Descriptive Sentences

**Figure 3.**
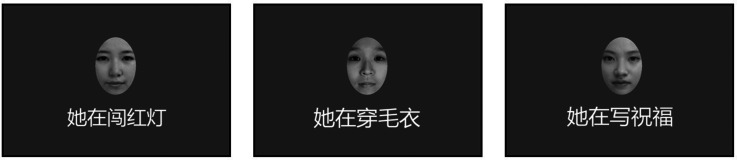
The examples of categories of stimuli used in this study. All of the behavior descriptive sentences were written in Chinese language. Each face was paired with a single behavior descriptive sentence from one of three categories: negative (left, “She is running a red light.”), neutral (middle, “She is knitting a sweater.”), or positive (right, “She is writing blessings.”).

We first collected 27 descriptive sentences of social behavior from three categories for this experiment (see examples in Figure 3): nine negative behaviors (NEG), nine neutral behaviors (NEU), and nine positive behaviors (POS). All the behaviors were based on everyday situations and referred to Chinese linguistic research. To better select the descriptive sentences, we asked another group of 16 volunteers (15 females and 1 male; mean age at 20.1) to rate the affective value of these behaviors on an 8-point scale (1 for most negative and 8 for most positive) (paradigm adapted from Rhodes & Jeffery, 2006; Ying et al., 2020). This pre-test was conducted online (https://www.wjx.cn/vj/tMKNZeY.aspx). Participants were asked to complete it by using the computer alone. Based on these ratings, we selected four behaviors for NEG (*M* = 2.56, *SD* = 0.528), NEU (*M* = 4.72, *SD* = 0.166), and POS (*M* = 6.69, *SD* = 0.331) as the affective person knowledge for the whole study.

##### Faces

There were 24 target faces and 12 memory faces used in this experiment (in total of 36 different identities, all female, all from Chinese ethnicity). All the neutral expressions were chosen from four face databases: the Nanyang Facial Emotional Expression Database (Yap et al., 2016), the Taiwanese Facial Expression Image Database (Chen & Yen, 2007), and the database used by Wang et al. (2015).

##### Procedure

This experiment includes three stages. In the first stage, participants were told that this was a memory experiment and instructed to memorize the behavior of a given face. If they could imagine the person is doing the behavior (from the descriptive sentence), they would have a better memory. Each face–behavior pair was presented for 4 s, modified from the research did by Todorov et al. (2007). After the stimuli disappeared, participants respond to the emotional valence of social behavior (1 for negative, 2 for positive). We set this task to keep participants’ attention and strengthen their memory. In this stage, for each participant, behaviors were randomly assigned to faces. Thus, the same face appeared with different behaviors for different participants. The order of face–behavior pairs was randomized. Each face–behavior pair was repeated 10 times. In the middle of 120 trials, participants would have a rest of 1 min.

**Figure 4.**
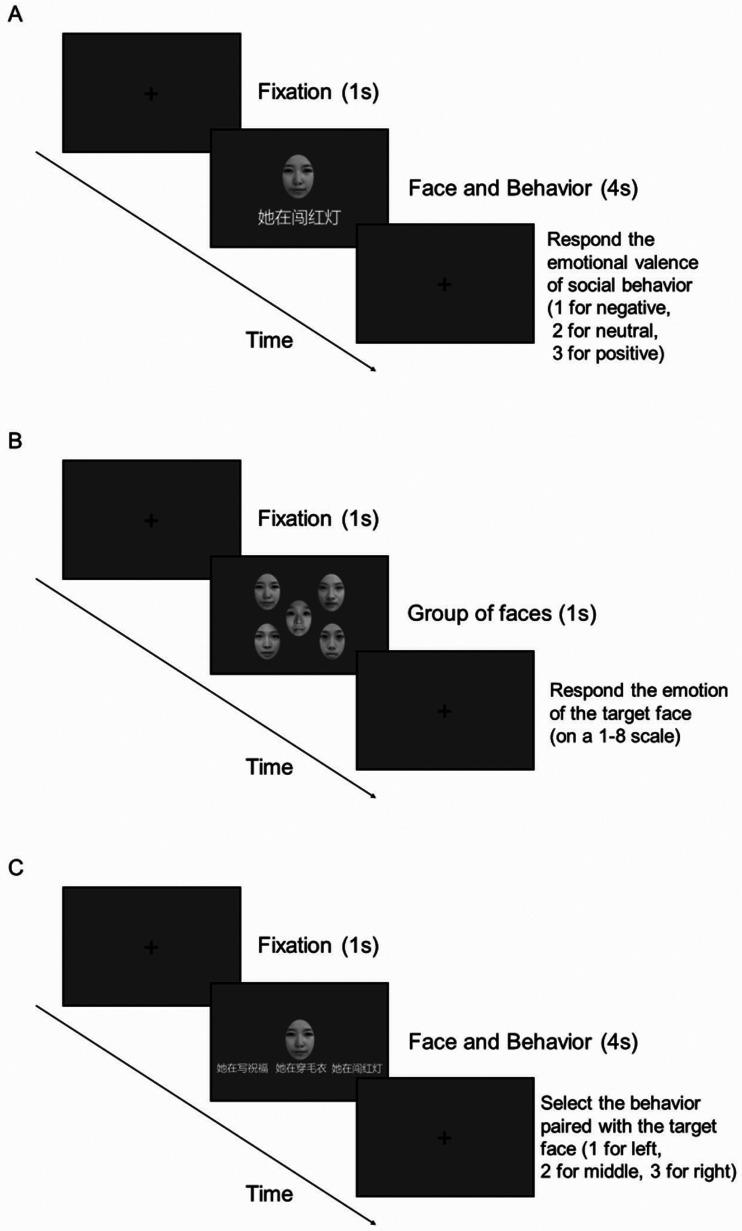
The procedure of experiment. (A) The memory task, (B) the main task (rate the emotional valence of the central target face), and (C) the memory validation task.

In the second stage (main task), there were four experimental blocks in randomized orders: (1) the baseline condition with only the target face (baseline), (2) the NEG condition where the target face was surrounded by four faces of negative behaviors, (3) the NEU condition where the target face was surrounded by four faces of neutral behaviors, and (4) the POS condition where the target face was surrounded by four faces of positive behaviors. The order of trials was randomized for each participant. During each block, each trial presented 1 of the 24 target faces randomly. The target face was presented in the center of the screen. Four faces of the same social–emotional surround the target face in random locations. Each target face was repeated three times. Each trial commenced with a 1-s interval (Figure 4B). After that, the target face appeared with (or without) the two flankers for 1 s. After they disappeared, the participants were asked to rate the target face on an 8-point scale (1 for most negative and 8 for most positive).

**Figure 5.**
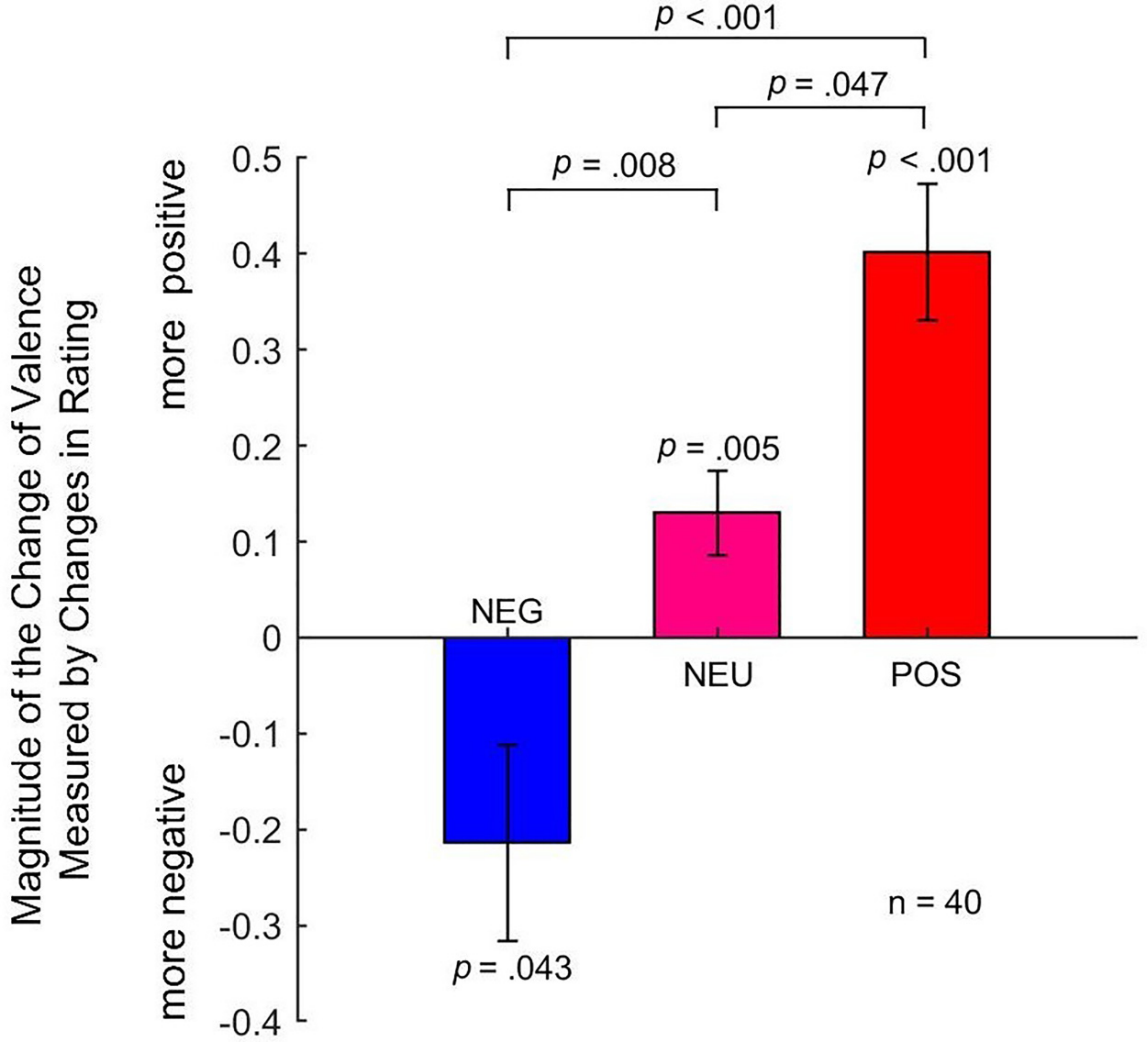
The summary of the Experiment 2 results. For the negative (NEG) condition, the perceived emotion of the target face was marginally decreased, while for the neutral (NEU) and positive (POS) conditions, the perceived emotional valence of the target face has been significantly increased. The magnitudes of the assimilation effect resembled the valence of each category of social behaviors. All *p* values of the pair-wise comparisons were with Bonferroni corrections.

In the third stage (memory validation task), participants were asked to recall the behavior paired with the face in the first memory task. Each of the 12 faces is repeated five times. Thus, there were 60 trials in this stage in total (Figure 5).

### Results

The results of one sample *t*-test suggested that at the negative [*M*_NEG_ = −0.214, *SEM* = 0.102, *t*(39) = −2.087, *p* = .043, Cohen's *d* = −0.330] condition, the perceived emotion of the target faces was biased to be more negative. At the neutral [*M*_NEU_ = 0.130, *SEM* = 0.044, *t*(39) = 2.973, *p* = .005, Cohen's *d* = 0.470] condition, the perceived emotion of the target faces was biased to be more positive. At the positive [*M*_POS_ = 0.402, *SEM* = 0.071, *t*(39) = 5.638, *p *< .001, Cohen's *d* = 0.891] condition, the perceived emotion of the target faces was biased to be more positive.

The results of repeated-measures ANOVA suggested that the three categories were significantly different (Greenhouse–Geisser corrected *F*(1.25,48.78) = 15.605, *p *< .001, *η*^2^_p_ = 0.286). Further pairwise comparisons with Bonferroni corrections suggested that differences between NEG and NEU conditions were significant (*p *= .008), differences between NEG and POS conditions were dramatically significant (*p *< .001), and differences between NEU and POS conditions were significant (*p *= .047).

The aim of the memory test task was to check whether the participant could remember the face and associated social behavior as a function of the memory task before. The results suggested that the identification of association [*M* = 53.4%, *SD* = 10.2%, *t*(39) = 12.451, *p* < .001, Cohen's *d* = 1.969] is significantly more accurate than random (one out of three social behaviors: 33.3%).

### Discussion

Here in Experiment 2, we conceptually replicated the first experiment and extended its finding. The results suggested that surrounding faces with different “artificial” emotional valence could affect the emotional perception of the target face differently. When the surrounding faces were of negative emotional valence (NEG), one would perceive the face as more negative than the baseline. When the surrounding faces were of neutral and positive emotional valence (POS), one would perceive the face as more positive than the baseline. Thus, the magnitudes of the facial emotion change were modulated by the emotional valence of the surrounding faces, which is a process of assimilation. Moreover, when the surrounding faces were of neutral emotional valence (NEU), the target face was also be perceived as slightly more positive, which could be explained by the “social positive effect”: mere presence of surrounding faces makes the target face look more positive. What's more, differences between the three experimental conditions (NEG vs. NEU, NEG vs. POS, NEU vs. POS) were all significant. Therefore, the emotional perception of the target face could be affected by emotional valence formed from affective personal knowledge on memories, which process is consistent with the comprise of both a “social positive effect” and an “assimilation” between the emotional valence of the surrounding faces and the target face.

## General Discussion

In this study, data from two experiments together suggested the perceived emotional valence of a face can be influenced by the emotional information of surrounding objects and faces, which is an assimilation process where the directions of change are roughly coincided with their levels of emotional valence. What's more, the neutral condition shows a social positive effect: mere presence of surrounding stimuli could make the target face look more positive. Thus, the way how surrounding stimuli affect the perception of target face is consistent with the existence of both (1) social positive aspect (being surrounded by other stimuli would *increase* the valence of the target face) and (2) assimilation aspect (an *assimilation* between the emotional valence of the surrounding stimuli and the target face). Moreover, not only objects with different emotional valence could affect face emotion perception; faces associated with social–emotional behaviors have the same impact. Therefore, the mechanism behind assimilation can not only be explained by low-level visual perception, certain high-level statistical processing is of high possibility.

Why does the emotion of the target face change alongside the emotional valence of the surrounding stimuli? It seems that Bayesian perception could partly explain this context-sensitive perception: probabilistic perceptual inference integrating prior experience and knowledge through top-down influences (Balas, 2013; Maier et al., 2022). For example, the possibility of pencils appearing in the kitchen is very small but much higher in the classroom. Similarly, it is possible for our brain to believe that positive faces should appear within a positive background. Therefore, the mechanism of these surrounding stimuli used in our research affecting the target face perception is possibly through prior experience, knowledge, or even belief (or bias). Previously, Wang et al. (2016) used a neurocomputational model of face and object processing to prove that there is a shared resource across face and object recognition, suggesting a common high-level visual cognition of domain-general ability where experience plays an important role in moderating (Gauthier et al., 2014). Thus, the perception and processing of the surrounding stimuli may be utilized to aid (and thus affect) face processing (Mohsenzadeh et al., 2019; Righart & de Gelder, 2008a).

Another explanation is that the “assimilation effect” may share some computational mechanisms with the summary statistics of emotional information across the target face and surrounding stimuli. The significant difference found between two conditions (UA vs. HO, UA vs. PF) in Experiment 1 and among three conditions (NEG vs. NEU, NEG vs. POS, NEU vs. POS) in Experiment 2 suggested that both emotional objects and faces in the context could affect the emotional perception of the target face. Through statistical processing (e.g., ensemble coding, serial dependence), our visual system can automatically utilize the essential information of the stimuli to better represent the target stimulus (Alvarez, 2011; Haberman & Whitney, 2007; Righart & de Gelder, 2006; Sama et al., 2021). Therefore, in the “assimilation effect,” participants also utilize the high-level emotional information of surrounding stimuli to perceive the target face. Future experiments should explore the similarity and the relationship between the “assimilation effect” and summary statistics.

In lots of real-world scenarios, faces appeared environments which provide a rich source of contextual associations (Oliva & Torralba, 2007). Faces have been widely shown to be more attractive when viewed with surrounding faces than alone in isolation (Carragher et al., 2019; Furl, 2016; Walker & Vul, 2014; also, by Ying et al., 2019 as “friend effect”). But the mechanism behind this effect found in these studies is still not fully clear. Some findings suggested that the face is more attractive to look with more attractive surrounding faces (e.g., Walker & Vul, 2014), but some found a reversed effect (e.g., Ying et al., 2019). But in general (e.g., Burns et al., 2021), researchers agreed that this effect is (at least partially) determined by the mean representation of the surrounding faces. What's more, Carragher et al. (2019) found that being surrounded by two houses (one on the left side, another on the right side) would also improve the attractiveness of the central face, which could be explained by the social positive effect proposed by Ying et al. (2019). Therefore, the perception of a face is actively affected by the surrounding stimuli whether they are objects or faces.

Similar to attractiveness, emotion is also an important part of face perception. Righart and de Gelder (2008a, 2008b) found that the perceived emotion of a face could be influenced by the emotional information of the background scene. However, direction of this contextual influence on facial emotional perception is still unclear. What's more, their stimuli were shown in the background scene, which means the target face was much smaller than objects if it was aimed to be put in the center of the background scene. Therefore, in this study, we modified the experimental design of Carragher et al. (2019), with the control of emotional valence of surrounding stimuli to further explore the effect of emotional stimuli in the context and their mechanisms.

The two experiments here used different kinds of stimuli for different purposes. In Experiment 1, we used different kinds of objects (houses, pleasant flowers, unpleasant flowers, and unpleasant animals) with different levels of emotional valence. Therefore, not only can we minimize the confounding factors like using different categories of stimuli (houses vs. animals), but we can explore what role of emotional valence plays in the process of facial perception (pleasant vs. unpleasant flowers). Moreover, people display a variety of social behaviors in their lives, and the value judgments of these behaviors can also elicit emotional responses from individuals, which are referred to as “social–emotional valence.” In Experiment 2, we used three groups of behavioral descriptive sentences. Through the memory task, faces will acquire the social–emotional valence of the corresponding behavior. It is important to note that, unlike the emotional valence of objects, the social–emotional valence of surrounding faces is based on memory. Therefore, together with the more rigorous experimental environments [in lab (Exp. 2) vs. online (Exp. 1)] and the fact that both target and flanker stimuli were faces, we were able to minimize the effects of visual attentional bias toward the flankers.

In Experiment 2, the target faces were surrounded by four other faces which have been “attached” with “artificial” emotional information (adapted from Todorov et al., 2007). This configuration of faces (surrounding and the target) resembles the “cheerleader effect” (e.g., Carragher et al., 2019; Walker & Vul, 2014; Ying et al., 2019) or “friend effect” (a term advocated by Burns et al., 2021). Because of the randomized stimuli selection procedure of the experiment (see “Methods” section for details), we think that the “cheerleader/friend effect” may not affect the interpretation of the data: when comparing the impact of the attached emotional information between conditions, the “cheerleader/friend effect” has been canceled out as the fact that in each condition, the target face was surrounded by four randomly selected faces. On the other hand, we believe that the significant attractiveness boost in the “neutral” condition (which resembles classical “cheerleader/friend effect” experiments where there was no emotional manipulation) would offer new evidence for supporting the ubiquitous-ness of the “cheerleader/friend effect.” Future researchers should consider testing the contribution of emotional manipulation and emotional memory (e.g., Hsieh et al., 2021) in “cheerleader/friend effect.”

In these two experiments, we tested the contribution of surrounding stimuli to the target face. In other words, we tested the “spatial” influence. Recently, researchers are also interested in testing the “temporal” aspect of facial attractiveness. For instance, many researchers tested the influence of the previous perception on the current perception: the serial dependence (e.g., Taubert et al., 2016). This effect has been found in many facial perception tasks (e.g., Yu & Ying, 2021). Although it sounds unlikely, Yu et al. (2023) using hidden Markov models suggested that the spatial and temporal influence the face perception in a complicated way. Therefore, it is worthwhile for future researchers to test our findings at the temporal aspect: “How would the pleasant-ness of flowers shown before a face impact the attractiveness of the face?”

In summary, this study provided evidence suggesting that different kinds of surrounding stimuli with different emotional valence could influence and modulate the perceived facial emotion differently: the positive surrounding stimuli will make an individual look more positive, while the negative stimuli would comparatively decrease the valence. Therefore, surrounding stimuli would induce an “assimilation effect” in line with their emotional valence, which is different from the contrast effect found in the attractiveness of group faces before (Ying et al., 2019), indicating that emotion and attractiveness are two different facial traits. Taken together, if individuals want to look positive in photos, they should surround themselves with beautiful flowers or kind people and avoid unpleasant animals or unkind people.
